# The complete mitochondrial genome of *Fannia scalaris* (Diptera: Muscidae)

**DOI:** 10.1080/23802359.2020.1820391

**Published:** 2021-05-31

**Authors:** Hui Xie, Liyang Sun, Jingjing Huang, Hua Wang, Yi Jiao, Jie Yan, Yaqun Guan

**Affiliations:** a Department of Forensic Science, School of Basic Medical Sciences, Xinjiang Medical University, Urumqi, P. R. China; b Department of Biochemistry and Molecular Biology, School of Basic Medical Sciences, Xinjiang Medical University, Urumqi, P. R. China; c Department of Forensic Science, School of Basic Medical Sciences, Central South University, Changsha, P. R. China

**Keywords:** Mitochondrial genome, *Fannia scalaris*, phylogenetic relationship

## Abstract

*Fannia scalaris* (Fabricius, 1794) is closely related to human life in ecological habits, which can lead to health concerns since they feed on various contamination sources. In this study, we first present the mitochondrial genome (mitogenome) of *F. scalaris* (GenBank No. MT017706). The length of this mitogenome was composed of 15,040 base pairs, including 13 protein-coding genes, two ribosomal RNA, 22 transfer RNA, and an AT-rich region. It consisted of A 39.3%, G 9.1%, C 13.0%, and T 38.6%. The arrangement of the genes was consistent with that of the ancestral metazoan. Furthermore, phylogenetic relationship indicated that *F. scalaris* was obviously separated from the muscid flies. This study provides useful genetic data in order to further understand the evolutionary relationship of the Muscidae.

*Fannia scalaris* (Fabricius, 1794) (Diptera: Muscidae) belongs to subfamily Fanniinae followed the morphological classification, which is closely related to human life in ecological habits and can lead to health concerns since they feed on various contamination sources (Xu and Zhao 1996), but it has not yet been studied in forensic medicine. It is widely distributed in many countries, including China, Japan, and North Korea (Wang et al. 2007). In this study, the length of mitogenome of *F. scalaris* was 15,040 bp (GenBank No. MT017706), containing 39.3%, G 9.1%, C 13.0%, and T 38.6%, with 13 PCGs, two *rRNA* genes, 22 *tRNA* genes, and one AT-rich region. The arrangement of all genes was conserved in the Muscidae, which was consistent with that of putative ancestral insect (Ren et al. 2019).

The adult specimens of *F. scalaris* were collected in June 2019 from Ürümqi city (43°50′ N, 87°37′E), Xinjiang province, China. All specimens were killed by freezing methods and then identified by the keys of external morphological characteristics (Xu and Zhao 1996). After that, these specimens were stored at −80 °C in Guo’s lab (Hunan, Changsha, China) with a unique code (CSU19111966). Total DNA was extracted from thoracic muscle tissues of a sample of an adult fly using QIANamp Micro DNA Kit (Tiangen Biotech Co., Ltd, Beijing, China) following the manufacture’s instruction (Ren et al. 2020). In addition, the total mitogenome of *F. scalaris* was sequenced as implemented on an Illumina HiSeq 2500 Platform. Mitogenome de novo assembly was carried out with MITObim version 1.9 (https://github.com/chrishah/MITObim) and SOAPdenovo version 2.04 (http://bioinf.spbau.ru/spades). The rough boundaries of each gene were initially identified using MITOS2 Web Server (Ren et al. 2020).

Phylogenetic analysis of *F. scalaris* and other 11 muscids species were performed using maximum likelihood (ML) method based on the 13 PCGs, and *Megaselia scalaris* (Diptera: Phoridae) was used as an outgroup (Figure 1). ML analysis was performed by IQ-TREE version 1.6.12 (http://www.cibiv.at/software/iqtree) (Ren et al. 2020). The phylogenetic analysis showed that the clade of *F. scalaris* was clearly separated from other muscids species. This study provided crucial genetic data for further studying on evolution analysis and species identification of muscid flies.

**Figure 1.**
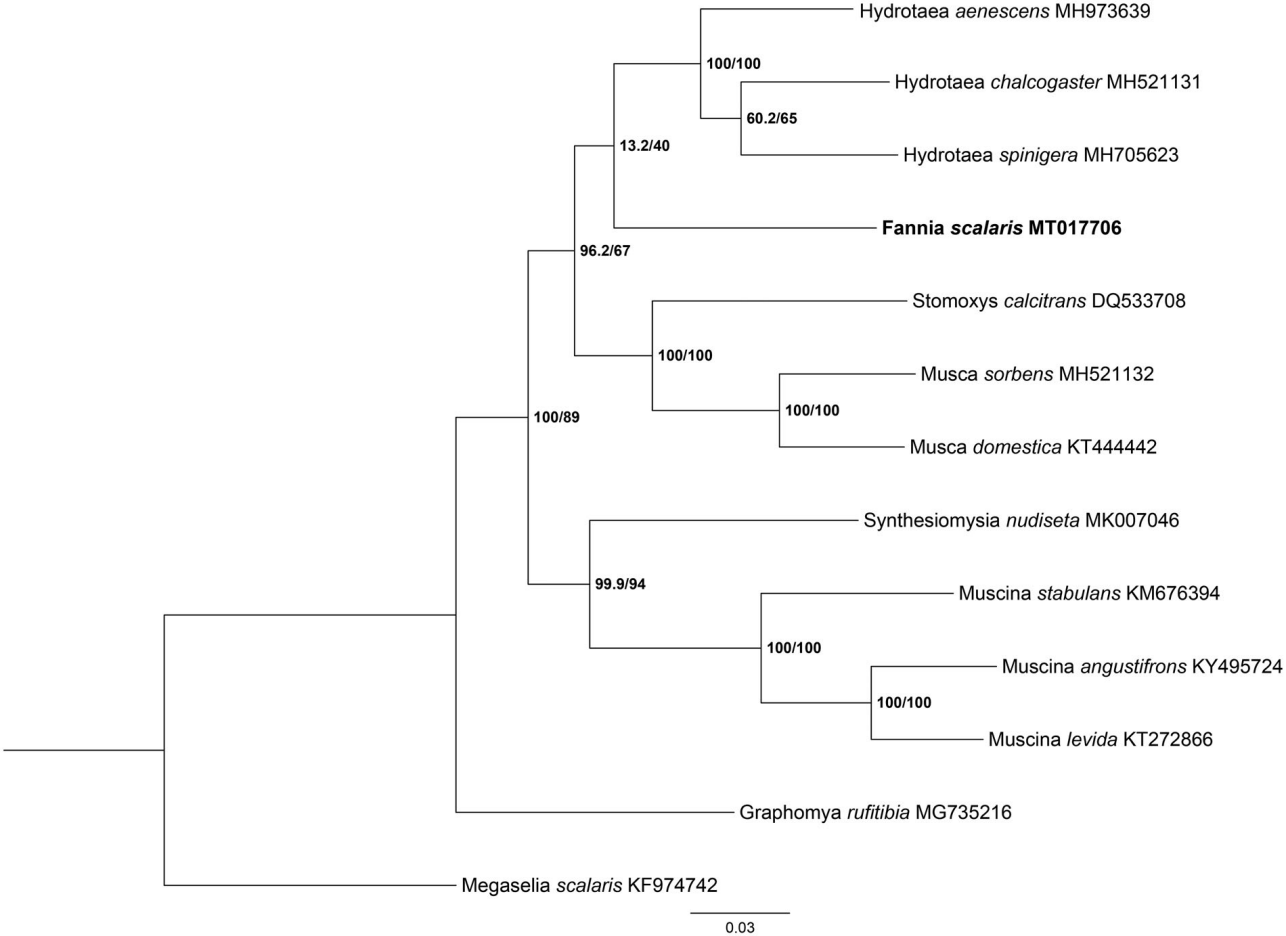
Phylogenetic tree of *F. scalaris* with 11 Muscidae species was constructed using maximum likelihood (ML) method based on the 13 PCGs. *Megaselia scalaris* was selected as an outgroup.

## Data Availability

The data that support the findings of this study are openly available in GenBank at https://www.ncbi.nlm.nih.gov (accession No. MT017706).

## References

[CIT0001] Ren L, Shang Y, Yang L, Shen X, Chen W, Wang Y, Cai J, Guo Y. 2019. Comparative analysis of mitochondrial genomes among four species of muscid flies (Diptera: Muscidae) and its phylogenetic implications. Int J Biol Macromol. 127:357–364. 30658142 10.1016/j.ijbiomac.2019.01.063

[CIT0002] Ren L, Zhang X, Li Y, Shang Y, Chen S, Wang S, Qu Y, Cai J, Guo Y. 2020. Comparative analysis of mitochondrial genomes among the subfamily Sarcophaginae (Diptera: Sarcophagidae) and phylogenetic implications. Int J Biol Macromol. 161:214–222. 32526299 10.1016/j.ijbiomac.2020.06.043

[CIT0003] Wang M, Liu L, Wang R, Xue W. 2007. Review of the *F. scalaris* species-group of the genus Fannia Robineau-Desvoidy, 1830 (Diptera: Fanniidae) from China. Pan Pac Entomol. 83(4):265–275.

[CIT0004] Xu WQ, Zhao JM. 1996. Flies of China. Shenyang, China: Liaoning Science and Technology.

